# Discovery of novel druggable binding pockets on SARS-CoV-2 PLpro *via* fragment screening and insights for structure-based inhibitor design

**DOI:** 10.1016/j.jbc.2026.113453

**Published:** 2026-08-14

**Authors:** Tingting Wu, Zhenghua Zhou, Xingyu Li, Weihua Wang, Haijiao Zhang, Wenming Qin, Qingjie Xiao

**Affiliations:** 1 National Facility for Protein Science in Shanghai, Shanghai Advanced Research Institute, Chinese Academy of Sciences, Shanghai, People's Republic of China; 2 University of Chinese Academy of Sciences, Beijing, People's Republic of China; 3 College of Life Sciences, Shanghai Normal University, Shanghai, People's Republic of China

**Keywords:** SARS-CoV-2 PLpro, fragment-based drug discovery, X-ray crystallography, protein–ligand interactions, structure-based inhibitor design

## Abstract

Coronaviruses are a large family of viruses capable of causing severe respiratory diseases, with recent outbreaks posing major threats to global public health. The SARS-CoV-2 papain-like protease (PLpro) is an essential viral enzyme involved in viral replication and host immune evasion, making it an attractive target for antiviral drug development. However, no PLpro-targeted drugs have yet been approved. Here, we employed a fragment-based drug discovery (FBDD) strategy combined with crystallographic screening and identified 23 fragment compounds with well-defined electron density. These fragments bind to seven distinct sites on PLpro and reveal multiple compound scaffolds and potential binding pockets through systematic structural analysis. Notably, Site 2 represents a newly identified pocket adjacent to the canonical substrate-binding site (Site 1) and shows strong potential for fragment linking to enable inhibitor optimization. Among them, Frag368 exhibited good enzyme inhibitory activity. Furthermore, based on structural similarities among the fragments, the FDA-approved drug 5-fluorouracil was identified as a ligand for Site 2. Biochemical assays confirmed that 5-fluorouracil exhibits inhibitory activity against PLpro. Collectively, these findings provide a robust structural framework for the rational design of PLpro inhibitors and support the development of novel antiviral therapeutics.

Coronaviruses are a family of enveloped positive-sense single-stranded RNA viruses that infect humans and a wide range of mammalian hosts, which primarily cause respiratory diseases. Over the past 2 decades, coronaviruses have triggered several major global public health crises, including the 2003 outbreak of Severe Acute Respiratory Syndrome Coronavirus (SARS-CoV), the 2012 outbreak of Middle East Respiratory Syndrome Coronavirus (MERS-CoV), and the emergence of the novel coronavirus SARS-CoV-2 in 2019 (1). Among these events, the COVID-19 pandemic caused by SARS-CoV-2 has led to sustained worldwide transmission and has profound impact on public health systems and socioeconomic stability (2). Although vaccines and therapeutic antibodies have effectively prevented COVID-19 or reduced disease severity, the emergence of viral variants, including Omicron, has reduced their protective efficacy. These challenges underscore the urgent need to develop antiviral drugs that target critical coronavirus proteins.

During SARS-CoV-2 replication cycle, the papain-like protease (PLpro), a cysteine protease, acts as a crucial regulatory factor in viral pathogenicity through a dual mechanism. First, PLpro specifically recognizes the conserved LXGG motif at the cleavage sites within viral polyproteins (nsp1/2, nsp2/3, and nsp3/4) and catalyzes their cleavage to release functional non-structural proteins (nsps), a step that is indispensable for viral genome replication (3). Second, PLpro exhibits deubiquitinating (DUB) and deISGylating activities by removing ubiquitin and ISG15 (interferon-stimulated gene 15) modifications from host immune-associated proteins. Through these activities, PLpro effectively suppresses type I interferon signaling and other innate immune responses (4, 5, 6, 7). The dual role in both viral replication regulation and host immune evasion makes PLpro a critical target for the development of anti-coronavirus therapeutics.

Nevertheless, the development of PLpro inhibitors faces three major challenges. First, the classical substrate binding region, comprising the S1/S2 sub-sites, exhibits a relatively shallow and constrained binding groove, and the catalytic triad (Cys111–His272–Asp286) lacks deep pockets that can readily accommodate high-affinity inhibitor binding (3). Second, the BL2 loop (residues 267–272) exhibits pronounced conformational flexibility, which destabilizes the substrate-binding site and reduces inhibitor engagement (8, 9). Third, the virus can easily acquire resistance to inhibitors that target a single binding site (10).

To overcome these limitations, we employed a structure-guided fragment-based drug discovery (FBDD) strategy. In this approach, we screened a fragment library with molecular weights ≤ 300 Da and determined the structures of fragment-target protein complexes using X-ray crystallography, which enabled identification of weak-affinity interaction hotspots (11). Compared with conventional high-throughput screening, FBDD provides two key advantages: the early-stage acquisition of precise binding modes can guide fragment-growing or fragment-linking optimization; and it facilitates the discovery of allosteric sites beyond canonical-binding pockets, thereby mitigating challenges associated with PLpro structural flexibility. By constructing a crystallographic screening system for the SARS-CoV-2 PLpro-C111S mutant (resolution, 1.85 Å), we systematically mapped the potential binding sites of PLpro. From a screen of 800 fragment molecules, we identified 23 fragments that exhibited well-defined electron density maps. These fragments occupy seven distinct binding sites on PLpro. Systematic analysis of the fragment-PLpro interactions revealed diverse chemical scaffolds and multiple potential binding modes, providing important mechanistic insights for subsequent inhibitor design.

Among these sites, Site 2 represents a newly identified binding pocket located adjacent to the classical substrate binding site (Site 1). Three-dimensional structure showed that Site 2 contains a conserved hydrogen-bonding network and presents a favorable topology for fragment engagement. Its close spatial proximity to Site 1 provides a clear structural basis for designing dual-site inhibitors that can bridge Site 1 and 2. Molecular dynamics simulations further demonstrated that linking fragments bound to Site 1 and Site 2 enhances the stability of the BL2 loop. In addition, guided by structural similarities among fragments occupying Site 2, we identified the FDA-approved drug 5-fluorouracil as a Site 2 binder. Biochemical assays confirmed its obvious inhibitory activity against PLpro. Collectively, these findings establish a robust structural framework that addresses key challenges in PLpro druggability and opens new avenues for the development of potent antiviral therapeutics.

## Results

### Discovery of potential binding sites *via* crystal-based fragment screening

Crystal quality is essential for successful crystal-based fragment screening. To obtain high resolution structures, we introduced a C111S mutation by substituting the cysteine at position 111 with serine. This modification enabled the resolution of the apo crystal structure to be stably improved to 1.85 Å. Although residue C111 is part of the catalytic triad (C111–H272–D286) and located in the active site, structural alignment of the wild-type protein (PDB code 7NFV) and the C111S mutant revealed a Cα RMSD of 0.286 Å over 269 aligned Cα atoms, indicating that the C111S mutation caused only minor structural perturbations to the overall protein structure (Fig. S1) (12).

We screened a total of 820 fragments and harvested 723 crystals. Among these, 667 crystals yielded high quality diffraction datasets with resolutions higher than 3.5 Å. From this dataset, we identified 23 fragment molecules with well-defined electron density maps, as shown in Figures S2–S6, corresponding to an overall hit rate of approximately 3%. Data quality metrics further demonstrate the stability and reliability of the screening pipeline. Most datasets exhibited diffraction better than 2.5 Å, with completeness values exceeding 99%, and showed R_merge_ values centered around 0.2 (Fig. S7). Together, these results confirm that the crystallographic fragment screening strategy is stable, reproducible, and highly effective for identifying PLpro-binding fragments.

**Figure 1:**
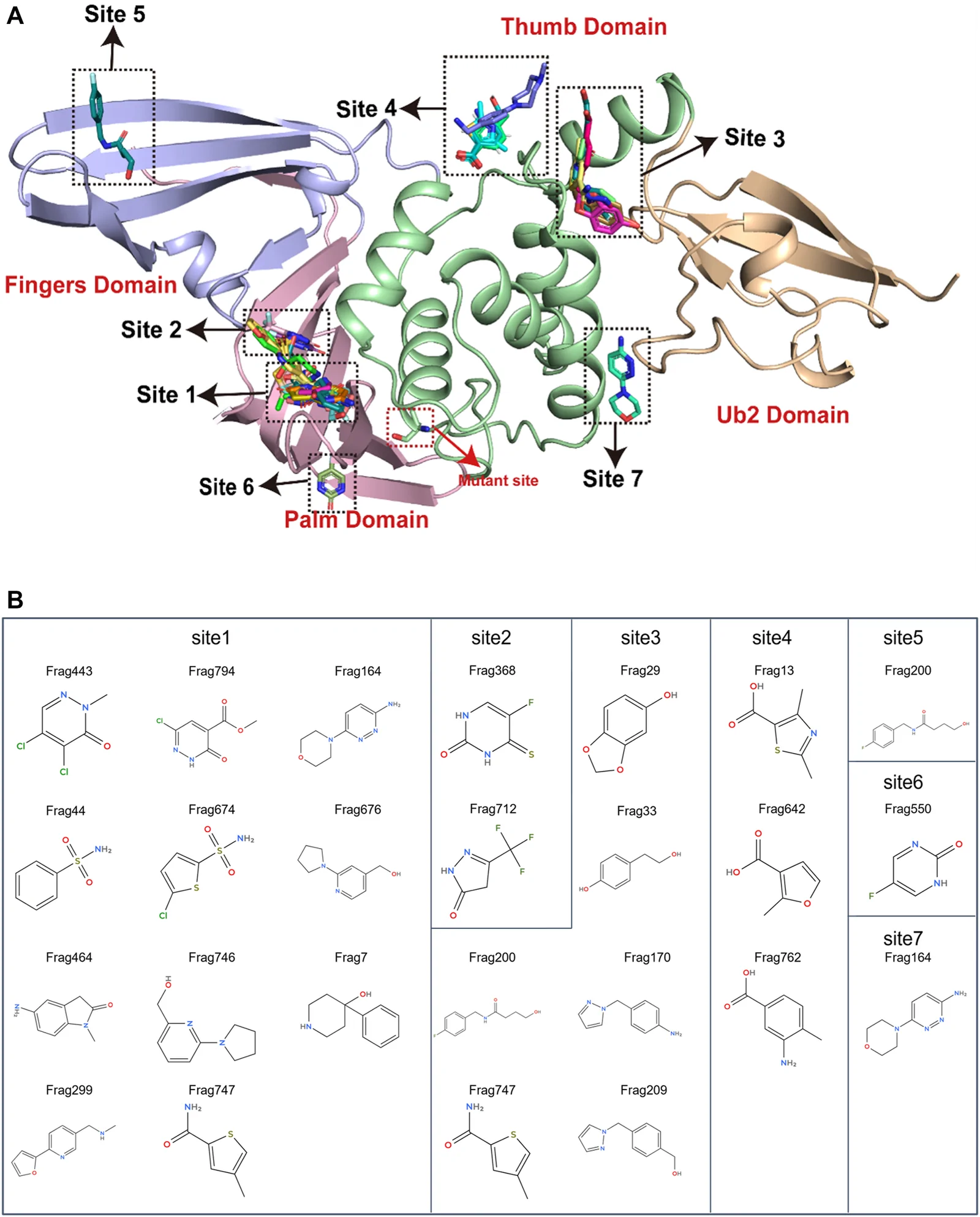
**Structural overview of fragment-binding sites in PLpro and chemical structures of the corresponding fragments.***A*, overall structure of PLpro showing the spatial locations of the fragment-binding sites and the bound fragments. Seven distinct fragment-binding sites (Sites 1–7) are highlighted by *black* dashed boxes. Domains of PLpro (Fingers Domain, Palm Domain, Thumb Domain, and Ub2 Domain) are indicated in different colors and labeled in red. Location of the introduced C111S mutation site is highlighted by the *red* dashed box. *B*, two-dimensional chemical structures of the 23 selected fragments grouped according to their crystallographic binding sites.

Structural alignment of all fragment-bound complexes revealed that the fragments occupy seven distinct binding sites on PLpro (Fig. 1). Specifically, 13 fragments bind to Site 1, 2 fragments bind to Site 2, 6 fragments bind to Site 3, and 4 fragments bind to Site 4, while Sites 5, 6, and 7 each accommodate one fragment. Notably, several fragments simultaneously engage more than one binding site. The identification of these diverse binding sites provides a structural basis for developing innovative and highly effective PLpro inhibitors.

### Binding characteristics analysis of Site 1 fragment molecules

To characterize the binding properties of the identified fragments and to establish a structural basis for small-molecule design, we performed a detailed analysis of the different binding sites. We used the PLIP web tool (https://plip-tool.biotec.tu-dresden.de/plip-web/plip/index) to assist in the systematic identification and classification of protein–fragment interactions.

We first examined the binding characteristics of fragments occupying Site 1, which corresponds to the LXGG substrate recognition site (13). Previous studies have shown that many small molecule inhibitors preferentially bind to this region (14, 15). By comparing shared interaction features among these fragments, we classified their binding behaviors into distinct binding modes.

**Figure 2:**
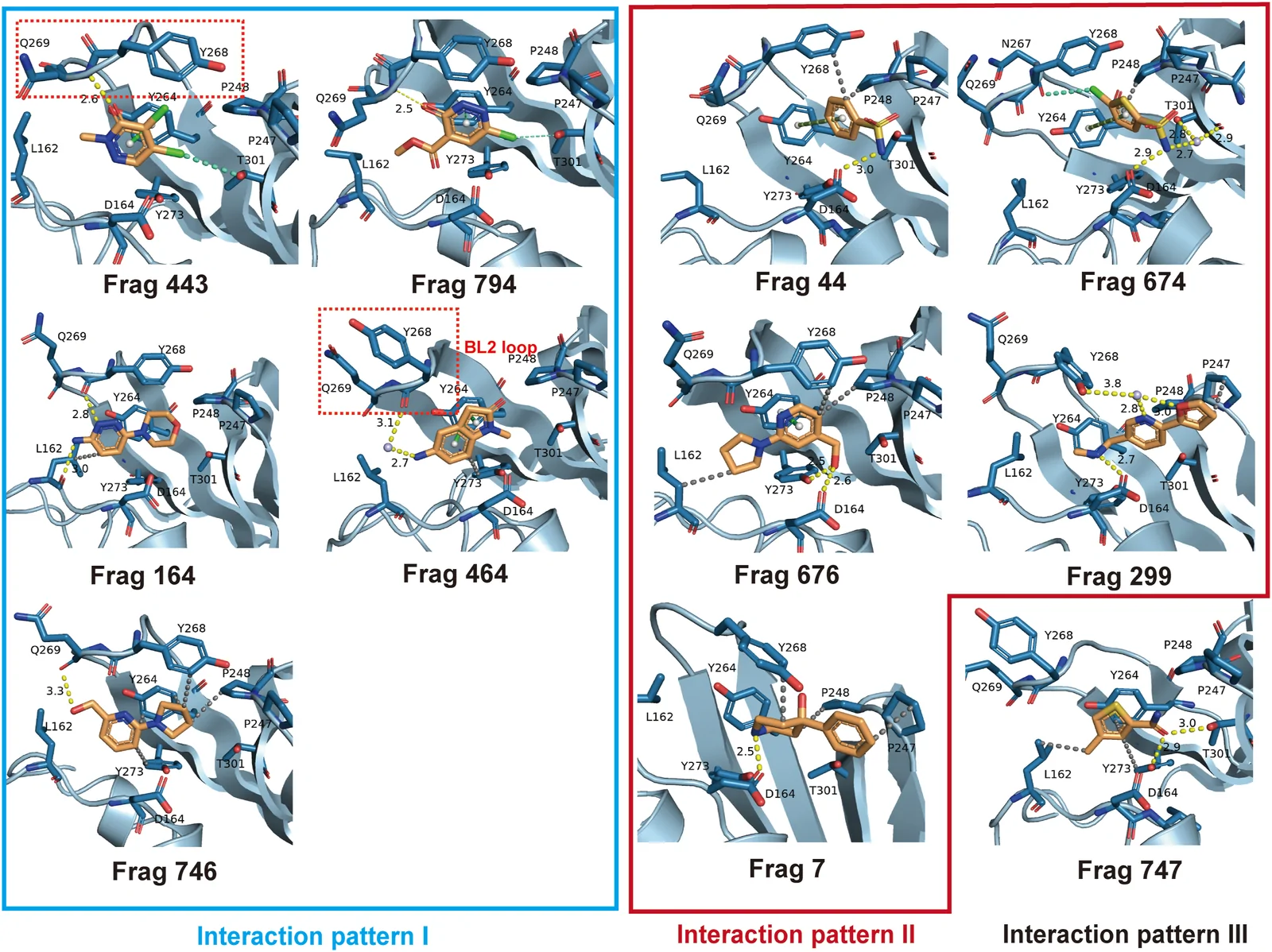
**Interactions between fragment molecules and PLpro-C111S at binding Site 1.** Hydrogen bonds are represented by *yellow* dashed lines, hydrophobic interactions are represented by *gray* dashed lines, halogen bonds are represented by *cyan* dashed lines, and π-π/T-shaped π interactions are represented by *green* dashed lines. *Red* dashed boxes highlight the BL2 loop region (residues Y268 and Q269), demonstrating the flexibility of the carbonyl oxygen on Y268 and the nitrogen atom on Q269. These can adopt flipped orientations to serve as either hydrogen bond donors or acceptors in different binding modes. The *blue* solid box denotes Interaction pattern I and the *red* solid box denotes Interaction pattern II.

In the first binding mode, backbone atoms of residues Q269 and Y268 within the BL2 loop (Blocking Loop 2) provide key hydrogen-bonding interactions (Fig. 2). In this mode, the main-chain nitrogen or oxygen atoms of Q269 and Y268 act as hydrogen bond donors or acceptors for these fragment molecules. Specifically, Frag443 and Frag794 form hydrogen bonds between the backbone nitrogen of Q269 and oxygen or nitrogen atoms on the fragments. In addition, the chlorine atoms of Frag443 and Frag794 form halogen bonds with the oxygen atom of T301. Frag164 and Frag464 engage the backbone oxygen atom of Y268 as a hydrogen bond acceptor, whereas Frag746 uses the backbone oxygen atom of Q269 as a hydrogen bond acceptor. Concurrently, residue Y264 forms π–π stacking interactions with the aromatic rings of these fragments, further stabilizing their binding (Fig. 2). Further structural analysis revealed that the carbonyl group of the Y268 main chain and the nitrogen atom of Q269 can adopt alternative orientations, allowing them as either hydrogen bond donors or acceptors depending on the binding mode. This flexibility is exemplified by the distinct interactions of Frag443 and Frag464 (Fig. 2, red dashed box).

The second binding mode is characterized by hydrogen-bonding interactions between the side-chain oxygen atom of D164 and the fragment molecules. Specifically, Frag44, Frag674, Frag676, Frag299, and Frag7 all form hydrogen bonds with D164. In addition, Frag44 and Frag674 engage residue Y264 through T-shaped π (T–π) interactions. Frag676 forms π–π stacking interactions, while residues Y268 and P248 further contribute hydrophobic contacts that stabilize the fragment within the binding pocket. Although Frag299 and Frag7 also establish hydrogen bonds with D164, they bind at the distal end of the active pocket, which limits the contact surface between their cyclic moieties and Y264. Instead, these fragments localize closer to residue P247, which provides a predominantly hydrophobic environment. Notably, Frag747 displays a distinct binding pattern compared with the other fragments, as it interacts with the side-chain oxygen atoms of Y273 and T301 (Fig. 2).

Analysis of the summarized fragment binding modes highlights the central role of Y264 in mediating aromatic ring interactions. In parallel, the presence of multiple hydrogen-bonding interactions creates a flexible and accommodating binding environment that allows fragments to occupy an expanded binding volume. The observed differences in fragment binding positions provide a clear structural basis for fragment linking, thereby enabling the rational design of small molecules with enhanced binding affinity.

### Binding characteristics analysis of Site 2 fragment molecules

**Figure 3:**
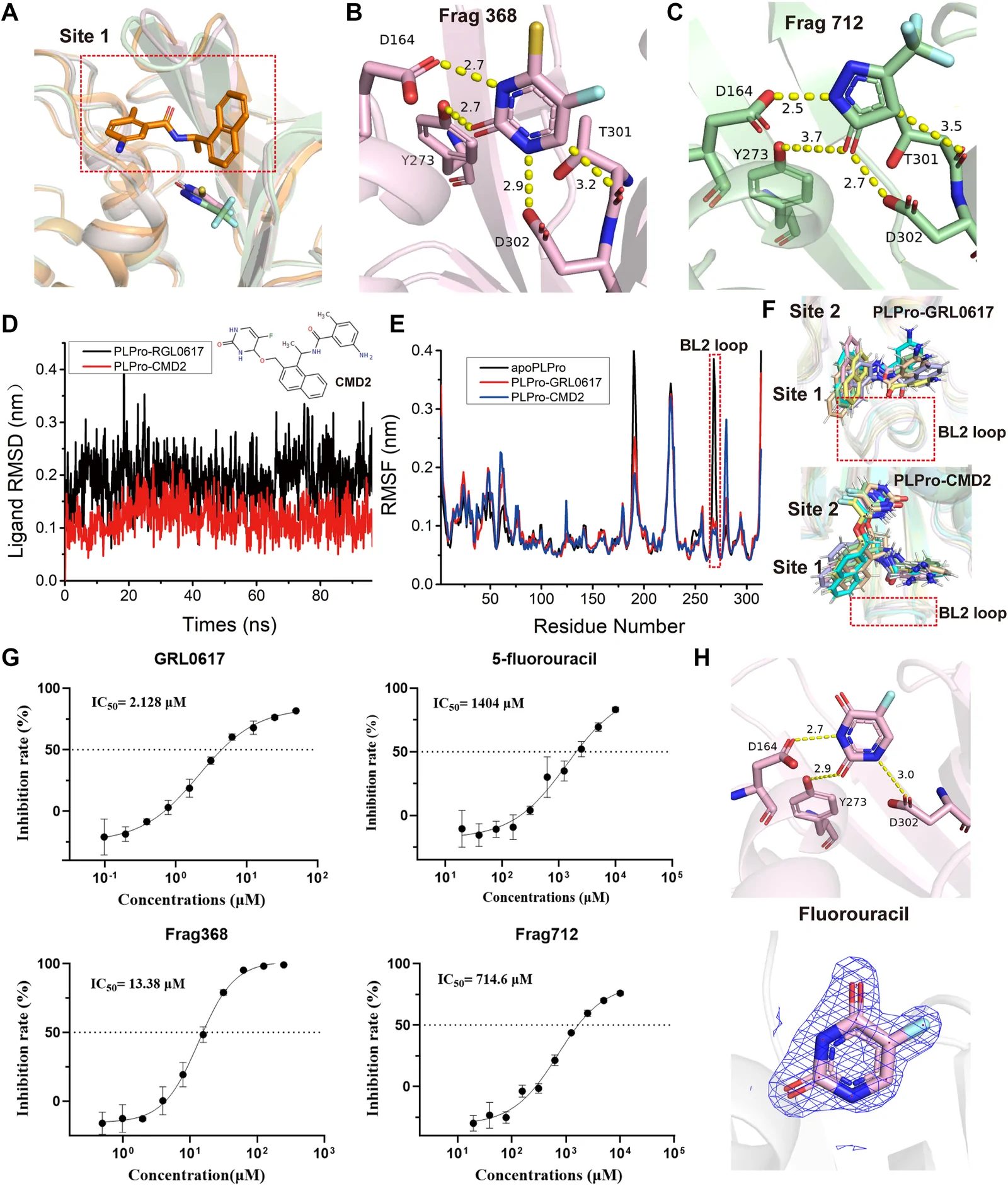
**Interactions between PLPro-C111S and Fragment Molecules at binding Site 2.***A*, overlay of Frag368 and Frag712 with the structure from PDB entry 7CJM. *B*, detailed interaction diagram showing Frag368 binding with PLPro. *C*, detailed interaction diagram showing Frag712 binding with PLPro. *D*, MD simulations of PLpro–CMPD-2 complex and PLpro–GRL0617 complex. *E*, MD simulations of BL2 loop in PLpro–CMPD-2 complex and PLpro–GRL0617 complex. *F*, Interaction diagrams of the ligand with PLPro at 0 ns, 20 ns, 40 ns, 80 ns, and 100 ns. Region highlighted by the *red* rectangle corresponds to the BL loop. *G*, Dose–response curves and IC_50_ determination of Frag368, Frag712, and FDA-approved compounds. GRL0617 was used as a positive control. Error bars indicate standard deviations (n = 3). *H*, structural basis of Site 2 binding. *Left*: Hydrogen-bond interactions between Frag368 and residues D164, Y273, and D302. Right: 2Fo–Fc electron density map (1.0 σ) confirming binding pose of Frag368/5-FU at Site 2.

Adjacent to the established Site 1, we identified a previously underexplored ligand-binding pocket, designated Site 2 in this study. Structural alignment of our fragment-bound structures with the previously reported PLpro–GRL0617 complex, in which GRL0617 occupies Site 1 (PDB code7CJM), revealed that Site 2 is located beneath and adjacent to Site 1 (Fig. 3*A*). To further confirm the structural independence of these two sites, co-soaking experiments were performed using frag164 or frag464 in combination with frag768.The resulting electron density maps displayed well-resolved and spatially distinct densities corresponding to fragments bound at Site 1 and Site 2 (Fig. S8). The presence of two spatially non-overlapping ligand densities strongly supports the notion that Site 1 and Site 2 are independent binding pockets that can be occupied simultaneously on PLpro. The fragments (frag368, frag712) form hydrogen bonds with the side-chain oxygen atoms of D164, Y237, and D302 (Fig. 3, *B* and *C*). Sequence alignment of PLpro from SARS-CoV, SARS-CoV-2, and MERS-CoV revealed that these three hydrogen-bond–forming residues are highly conserved across all three viruses (Fig. S9). This high level of conservation suggests that the identified compounds may also bind to PLpro from SARS-CoV, SARS-CoV-2, and MERS-CoV. Owing to the close spatial proximity of Site 1 and Site 2, chemical linkage of compounds targeting both sites may provide a viable strategy for the development of hybrid molecules with increased binding affinity and improved stability.

Based on this concept, we designed a hybrid compound (CMPD-2) by chemically linking a known Site 1–binding molecule (PDB code 7CJM) (16) with the identified Site 2–binding fragment (Frag368). To rigorously assess the hypothesis that anchoring an additional fragment at Site 2 enhances binding stability, molecular dynamics (MD) simulations with a duration of 100 ns were conducted for apo PLpro, the PLpro–GRL0617 complex, and the PLpro–CMPD-2 complex. Ligand root-mean-square deviation (RMSD) values after aligning protein backbone Cα atoms shows that the CMPD-2 complex exhibited significantly reduced ligand fluctuations (∼1 Å), whereas the PLpro–GRL0617 complex displayed larger fluctuations (∼2 Å), indicating more stable ligand binding in the CMPD-2 complex (Fig. 3*D*). We further evaluated root-mean-square fluctuation (RMSF) values of Cα atoms within residues forming the BL2 loop, both GRL0617 and CMPD-2 reduced BL2 loop flexibility relative to apo-PLpro, whereas CMPD-2 produced a markedly stronger stabilizing effect (Fig. 3*E*). To directly visualize these stability differences, we extracted representative structures at 20 ns intervals throughout the simulations. Compared with PLpro–GRL0617, both the ligand and the BL2 loop in the PLpro–CMPD-2 complex showed minimal conformational fluctuations over time (Fig. 3*F*). Together, these MD simulation results support the conclusion that anchoring a fragment at Site 2 reduces BL2 loop flexibility and enhances overall substrate binding stability.

To further explore scaffold-based drug repurposing, we screened more than 3000 FDA-approved compounds using the Frag368 scaffold as a search template. Following the primary screen, 5-fluorouracil was selected for secondary validation. Site 2 is located adjacent to the substrate binding region, thus, ligand engagement at this site is likely to directly affect PLpro catalytic activity. Then, we performed enzymatic inhibition assays using the well-established PLpro inhibitor GRL0617 as a positive control to ensure assay reliability (Fig. 3*G*). As shown in Figure 3*G*, all compounds exhibited concentration-dependent inhibition. The measured IC_50_ value of GRL0617 was 2.128 μM, which is consistent with previous reports. The IC_50_ values of Frag368, Frag712, and 5-fluorouracil were 13.38, 714.6, and 1404 μM, respectively. Frag368 showed relatively strong inhibitory activity against PLpro. Although it was less potent than GRL0617, its potential as a starting point for further structural optimization and fragment growing should not be overlooked. In addition, the co-crystal structure confirmed that 5-FU binds at Site 2 in an orientation closely resembling that of Frag368 (Fig. 3*H*), thereby validating the key pharmacophore features of this newly identified pocket.

Together, these results demonstrate that structure-guided, scaffold-based repurposing of FDA-approved drugs provides an efficient approach for identifying novel PLpro inhibitors and for validating previously unexplored allosteric sites, particularly in the context of emerging infectious disease outbreaks.

### Binding characteristics analysis of Site 3 fragment molecules

Previous studies identified three phenolic compounds that bind to Site three and inhibit the deISGylation of interferon-stimulated gene 15 (ISG15), thereby disrupting its interaction with PLpro (12). Given that ISG15 deISGylation is a key mechanism by which coronaviruses suppress host innate immune responses, site three represents an important and therapeutically relevant target.

**Figure 4:**
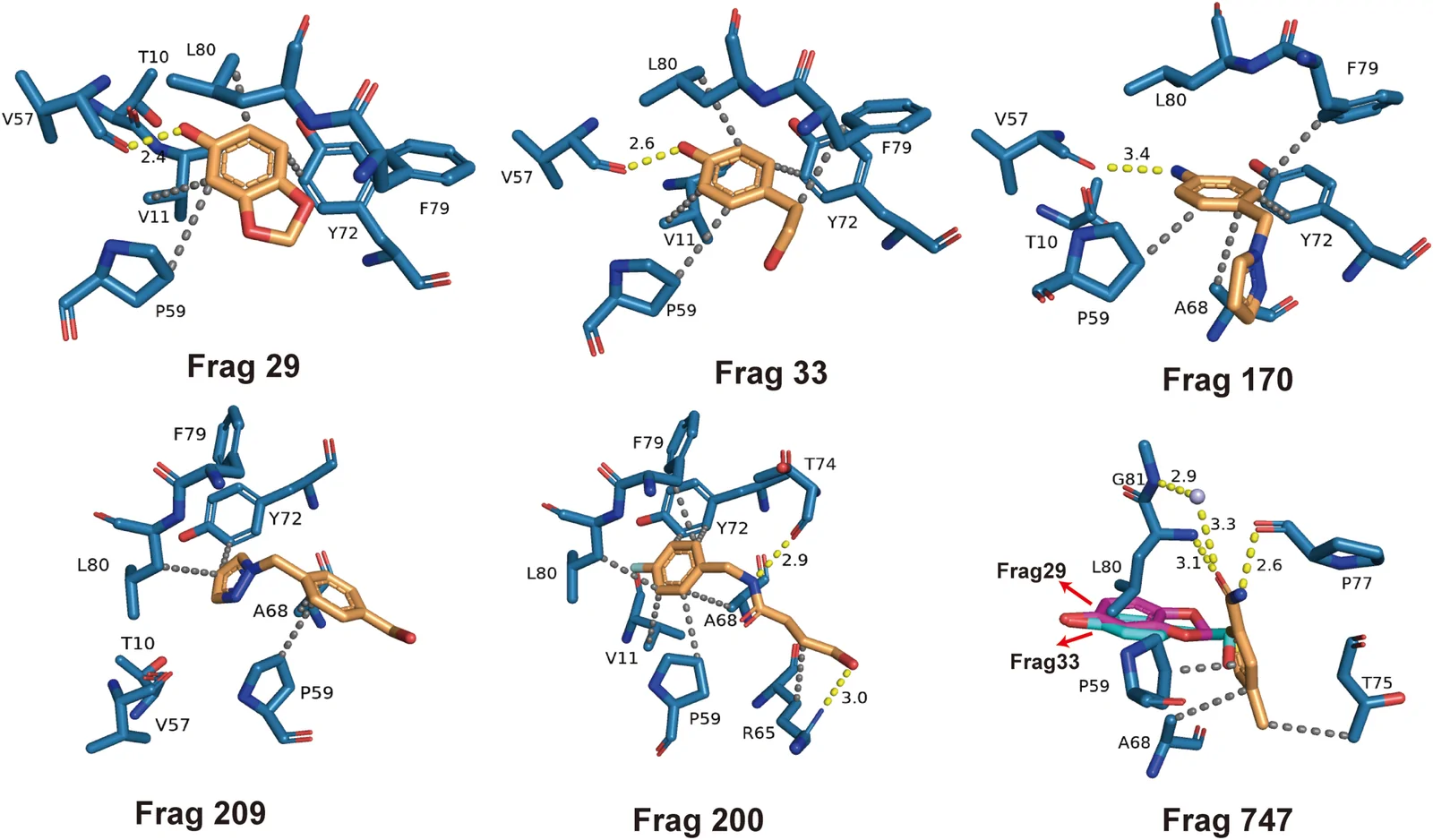
**Interactions between PLPro-C111S and Fragment Molecules at binding Site 3.** Hydrogen bonds are represented by *yellow* dashed lines, and hydrophobic interactions are represented by *gray* dashed lines.

Through fragment library screening, we identified several previously unreported fragment molecules that bind to Site 3, as shown in Figure 4. Statistical analysis of their binding modes revealed that residues V11, P59, L80, Y72, A68, and F79 form a hydrophobic environment that strongly supports fragment binding (Fig. 4). In addition to these hydrophobic interactions, the backbone carbonyl oxygen of V57 serves as a potential hydrogen-bond acceptor. Consistent with this role, proton-donating groups in Frag29, Frag33, Frag170, and Frag768 form hydrogen bonds with this backbone atom.

Notably, Frag170 and Frag209 display high structural similarity, with Frag170 bearing an amino substituent on the aromatic ring and Frag209 containing a hydroxymethyl group. Despite this similarity, the two fragments adopt binding orientations that differ by nearly 180 degrees. The binding modes of Frag29 and Frag33 suggest that this site may preferentially accommodate a hydroxyl group or a substituent of comparable size.

In contrast to the other fragments, Frag747 exhibits a distinctly different binding mode. Its aromatic ring does not fully insert into the hydrophobic pocket formed by V11, P59, V57, L80, and Y72. Instead, Frag747 binds in a unique orientation in which its aromatic plane is nearly perpendicular to those of Frag29 and Frag33.

Together, these observations reveal substantial binding diversity at Site 3 and provide important structural insights for structure-based drug design. Further optimization of these fragment scaffolds may enable the development of more potent PLpro inhibitors capable of disrupting coronavirus immune evasion mechanisms.

### Binding characteristics analysis of Site 4 and other sites fragment molecules

**Figure 5:**
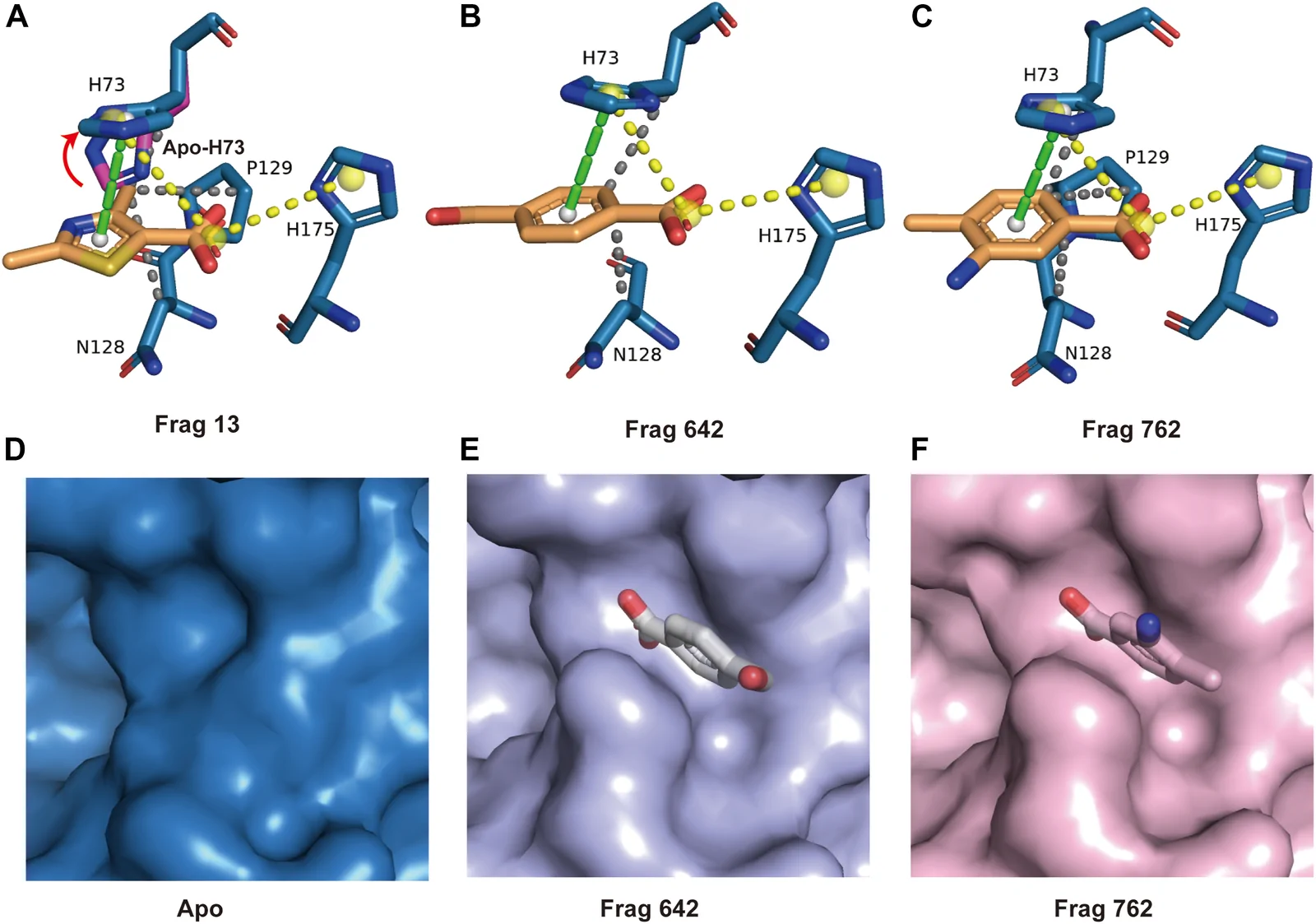
**Binding interactions between PLpro-C111S and fragment molecules at Site 4.***A*, structural alignment of the PLpro-C111S–Frag13 complex and the apo structure, showing a conformational shift in the H73 side chain upon fragment binding (*red arrow*). *B*, binding mode of Frag642 within the Site 4 pocket. *C*, binding mode of Frag762 within the Site 4 pocket. (*D*) Surface view of the apo structure. *E*, Ssurface view of PLpro-C111S with Frag642 bounded. *F*, surface view of PLpro-C111S with Frag762 bounded. Hydrogen bonds or electrostatic interactions are represented by *yellow* dashed lines, hydrophobic interactions are represented by *gray* dashed lines, and π–π/T-shaped π interactions are represented by *green* dashed lines. In the stick representations, oxygen atoms are colored *red* and nitrogen atoms are colored *blue*.

At Site 4, we identified three fragment molecules that bind clearly to this region. Compared with the apo structure, fragment binding induces a pronounced local conformational change at residue H73. Specifically, the H73 side chain undergoes an approximate 70° rotation, which generates a previously unobserved pocket that accommodates the fragments (Fig. 5*A*). Electrostatic surface representations of PLpro-C111S further illustrate the formation of this pocket upon fragment binding (Fig. 5, *D*–*F*).

At the molecular level, the carboxyl groups of the fragments form electrostatic interactions with residue H175, while the aromatic rings of the compounds participate in π–π stacking interactions with H73 (Fig. 5, *A*–*C*). In addition, nonpolar regions of the fragments establish hydrophobic contacts with the side chains of H73, P129, and N128, collectively contributing to the shape and stabilization of the newly formed hydrophobic pocket. Together, these observations demonstrate that fragment binding at Site 4 relies on coordinated conformational rearrangement, electrostatic interactions, and hydrophobic contacts. This combination of features suggests that Site 4 represents a viable and adaptable target for further fragment optimization and compound development.

In addition to the previously reported binding sites, we identified several additional fragment-binding regions on PLpro (Fig. S10). At Site 5, Frag200 associates with the protein predominantly through hydrophobic interactions. At Site 6, Frag550 forms a covalent interaction with residue C284. In parallel, residues S293 and T291 establish hydrogen bond interactions with nitrogen atoms on the fragment’s aromatic ring. Compared with the WT structure, fragment binding at Site 6 triggers pronounced local conformational rearrangements, including a marked displacement of Y296 that opens a pocket to accommodate the fragment. At Site 7, no major local conformational changes are observed compared with the WT structure; nevertheless, the fragment binds through hydrogen bond interactions with the side-chain atoms of D37 and N146.

## Discussion

FBDD using macromolecular crystals is a well-established and powerful approach for identifying novel molecular interactions and revealing druggable binding pockets. To date, more than 60 FBDD-derived compounds have advanced to clinical trials, and eight drugs have received regulatory approval, highlighting the success of this strategy (17). Structure-based fragment screening offers a key advantage because it enables direct visualization of fragment binding modes. This capability is particularly important for detecting weak-affinity fragments and identifying allosteric sites that are often challenging to observe using biochemical or biophysical methods alone.

Successful crystallographic fragment screening critically depends on the ability to grow highly stable crystals and to preserve crystal integrity during compound soaking, as both factors directly influence diffraction quality and accurate hit identification. In this study, we used a highly soluble fragment library dissolved in water, thereby avoiding DMSO or other organic solvents that frequently destabilize protein crystals. Prior to soaking, we pre-mixed fragment solutions with reservoir buffer at a 1:1 ratio to maintain crystallization conditions. This approach substantially improved crystal stability during soaking. Using this optimized strategy, we previously conducted a large-scale screen against the Pin1 protein and identified approximately 50 fragment hits, demonstrating the robustness and efficiency of the workflow (18).

PLpro has been extensively investigated as a target for structure-based antiviral drug discovery, and a large number of apo and ligand-bound structures have been deposited in the PDB. Previous studies have established several ligand-binding regions, most notably the noncovalent inhibitor-binding groove adjacent to the BL2 loop and the catalytic region surrounding Cys111 (16, 19, 20). The structural characterization of GRL0617 and related compounds provided an important foundation for subsequent inhibitor optimization, while more recent structure-guided studies have substantially improved inhibitor potency and pharmacological properties. For example, Garnsey *et al.* reported the development of the potent, selective, and orally available inhibitor PF-07957472, whose binding mode rationalized its enhanced activity relative to earlier PLpro inhibitors, and which showed efficacy in a mouse-adapted SARS-CoV-2 infection model (21).

In this work, fragment screening against PLpro revealed multiple binding sites that encompass several previously characterized ligand-binding regions. Importantly, our screen independently identified two fragment-binding regions, designated Site 2 and Site 4 in this study and enabled their detailed structural and functional characterization. Among them, Site 2 is particularly notable. It lies adjacent to the well-characterized Site 1 yet forms an independent and well-defined pocket. Structural comparisons and fragment co-soaking experiments demonstrated that Sites 1 and 2 can be occupied simultaneously, providing a structural basis for the design of dual-site ligands. While this manuscript was under review, Wang *et al.* independently reported a crystallographic fragment screen of SARS-CoV-2 PLpro, identifying 129 fragment binders distributed across 12 binding sites, including two fragments with micromolar inhibitory activity (22). Our study provides complementary mechanistic insights by demonstrating the simultaneous occupancy of the canonical Site 1 and the adjacent pocket designated Site 2, evaluating a Site 1–Site 2 fragment-linking strategy, and validating Frag368 and 5-fluorouracil as Site 2-binding inhibitors. Together, these complementary findings further expand the ligandable landscape of PLpro and provide distinct structural starting points for inhibitor development.

By analyzing multiple fragments bound at shared sites, we identified common interaction features as well as distinct binding modes, thereby establishing a robust structural framework for subsequent small-molecule optimization. For example, fragments identified at Sites 1 and 3 exhibited diverse interaction patterns, enabling us to define key residues essential for fragment binding and to derive valuable guidance for inhibitor design. Notably, several fragment compounds, including Frag200 and Frag747, showed the ability to bind multiple sites simultaneously. Specifically, Frag747 bind not only to Site 1 but also to Site 3. Previous studies have shown that simultaneous engagement of these two sites can block substrate binding and effectively inhibit the interaction with ISG15, thereby reducing deISGylation activity (12). Small molecules that target multiple binding sites may therefore display enhanced antiviral activity. Fragment library screening has also demonstrated strong potential for investigating interactions between a single compound and multiple biological targets. By querying the PDB database (https://www.rcsb.org/chemical-sketch), we identified several fragment molecules that bind to distinct proteins. For example, Frag44 binds to human carbonic anhydrase II (hCAII, PDB code 4JSA), human lipoprotein-associated phospholipase A2 (Lp-PLA2, PDB code 5JAD), and Zika virus RNA-dependent RNA polymerase (Zika Virus RNA-Dependent RNA Polymerase, PDB code 6LD3). Similarly, Frag13 binds to SARS-CoV-2 non-structural protein 3 (NSP3, PDB code 5RTK) and HIV-1 integrase (HIV-1 integrase, PDB code 3AO1). These observations demonstrate that fragment library screening can identify compounds with multi-target binding capability and can also provide an important reference framework for the design and optimization of multi-target therapeutics.

In addition, Symmetry analysis of the crystal packing revealed a pronounced interface between two PLpro molecules, with a buried surface area of 1488.4 Å^2^ as estimated by PDBePISA. In this arrangement, Site 1 lies in proximity to the symmetry-related copy of Site 3 (Fig. S11). Although PLpro is predominantly monomeric in solution based on size-exclusion chromatography, several fragments identified in our screen occupy Site 1 or Site 3 and partially overlap with regions of the symmetry mate. This geometry suggests a plausible strategy to design linked compounds that bridge Sites 1 and 3, and in turn stabilize a dimer-like assembly that may modulate PLpro function.

Frag124 binds at the interface near Site 3 (Fig. S11). A similar site was previously reported for fragment HE9 (PDB code 7OFU) (12), which shows antiviral activity. The interface location suggests that Frag124/HE9 may be better stabilized in a dimeric context than on an isolated monomer, raising the possibility that these ligands act by promoting or reinforcing PLpro–PLpro contacts. In this context, the PLpro dimer interface identified in this study represents a compelling target for the development of molecular glue–like inhibitors, which may enhance therapeutic efficacy by disrupting viral replication or by restoring antiviral immune responses. Structural superposition of one protomer of the Frag124-associated PLpro dimer with PLpro in the human ISG15 complex (PDB 7RBS) revealed that the second PLpro protomer occupies part of the space required for productive ISG15 recognition (Fig. S12*A*). The dimeric arrangement would therefore sterically restrict the binding of the two ubiquitin-like domains of ISG15 and interfere with the positioning of its C-terminal region toward the catalytic cleft. Thus, the proposed dimer may impair PLpro function by blocking productive macromolecular substrate recognition rather than by directly occupying the catalytic site. Molecular dynamics simulations showed that the crystallographically observed PLpro–PLpro arrangement remained stable throughout the 100 ns simulation, indicating that the dimer interface is dynamically feasible outside the crystal lattice (Fig. S12, *B* and *C*). Analysis of the protein-aligned RMSD showed that Frag124 remained relatively close to its crystallographically observed binding pose during the first approximately 15 ns, with an RMSD of around 2 Å. The ligand subsequently underwent pronounced positional and orientational rearrangements, transiently returned to an initial-like pose with an RMSD of approximately 2 Å, and then deviated again (Fig. S12, *D* and *E*). Throughout these transitions, Frag124 remained within the interfacial cavity, suggesting that it dynamically samples multiple configurations rather than maintaining a single rigid binding pose. Overall, the simulations support the structural stability of the PLpro dimer and reveal a dynamic interfacial binding mode for Frag124. Although these results do not establish persistent molecular-glue activity, they are consistent with the possibility that Frag124 transiently favors or stabilizes dimer-compatible conformations of PLpro.

To complement the structural characterization, we evaluated the enzymatic inhibitory activities of all 23 fragments for which crystal structures were obtained. All fragments were tested at 1 mM under the same assay conditions, given that unoptimized fragment hits may exhibit relatively weak inhibitory activity. The fragments displayed a broad range of inhibitory effects, with Frag368 showing the strongest inhibition and Frag712 also exhibiting substantial inhibitory activity, several fragments showed weak or moderate effects, whereas many fragments showed weak or negligible inhibition (Fig. S13). These results indicate that crystallographically observed fragment binding does not necessarily translate into measurable enzymatic inhibition,but such structurally characterized fragments may nevertheless provide useful starting points for subsequent fragment growing and structure-guided optimization. Although we get some good fragments, several limitations should be acknowledged. First, the identified fragments require further structural optimization to improve binding affinity and inhibitory potency. Second, although this study primarily relied on crystallographic evidence, additional biochemical and cellular studies are essential to validate the therapeutic relevance and antiviral efficacy of these fragment-derived inhibitors.

Overall, using crystallography-based fragment screening, we identified novel fragment hits and several previously unrecognized binding sites on SARS-CoV-2 PLpro. These structures expand the druggable landscape of PLpro and provide a framework for structure-guided inhibitor design. Further optimization and development of multitarget inhibitors based on these results hold strong potential for advancing therapeutic approaches against SARS-CoV-2 and its emerging variants.

## Experimental procedures

### Cloning, expression, and purification of SARS-CoV-2 PLpro

The PLpro gene sequence (corresponding to amino acids 746–1060 of non-structural protein 3, YP_009742610.1) was cloned into the pET21b vector and transformed into *Escherichia coli* BL21 (DE3) competent cells. To facilitate subsequent crystallization experiments, a C111S mutant was also constructed. The PLpro C111S mutant (PLproC111S) was generated using polymerase chain reaction (PCR). Positive clones were screened on solid medium containing ampicillin, and selected clones were inoculated into liquid medium. When the OD600 of the bacterial culture reached 0.6 to 0.8, 0.5 mM IPTG was added to induce protein expression, and the culture temperature was adjusted to 18 °C for overnight incubation. After incubation, the cells were harvested by centrifugation at 4000 rpm for 10 min, followed by cell lysis using ultrasonication in lysis buffer (150 mM NaCl, 20 mM HEPES, pH 7.5, 5 mM DTT). The cell lysate was stored at 4 °C and centrifuged at 12,000×*g* for 20 min to remove cell debris. The supernatant was subjected to Ni-NTA affinity chromatography for purification. PLpro was eluted from the Ni-NTA resin using lysis buffer containing 300 mM imidazole. For further purification, size-exclusion chromatography (SEC) was performed using a HiLoad 16/600 Superdex 200 column (GE Healthcare, GB) connected to an ÄKTA purification system. The column was equilibrated with 150 mM NaCl, 20 mM HEPES, pH 7.5, 5 mM DTT. After purification, peak fractions were collected. For subsequent crystallization experiments, PLpro-C111S protein was concentrated to approximately 20 mg/ml, aliquoted into 100 μl portions per tube, and rapidly frozen using liquid nitrogen for storage.

### Crystallization

Crystallization of the PLpro-C111S protein was carried out at 4 °C using the hanging drop vapor diffusion method. The crystallization condition consisted of 1.6 M sodium malonate dibasic monohydrate at pH 6.0. Crystals formed within 3 days. To ensure effective binding and stable crystal formation for soaking with approximately 800 small molecules, it was necessary to have crystals growing in at least 80% of the wells on the crystallization plate. Therefore, a seeding method was employed. Initial crystals were crushed and added to a crystallization solution containing 1.6 M sodium malonate dibasic monohydrate (pH 6.0). 1 μl aliquot of the seed crystals was added to 10 ml of the crystallization solution, which was then used as the reservoir solution. A mixture of 0.8 μl reservoir solution and 0.8 μl protein solution was prepared and stored at 4 °C. This method effectively ensured crystal formation in over 80% of the wells on the crystallization plate.

### Small molecule soaking

The fragment compound library was sourced from Toslab with the catalog number L7800, and the initial concentration of the small molecules was 100 mM. For the small molecule soaking procedure, 0.5 μl of the fragment compound was mixed with 0.5 μl of reservoir solution, and the mixture was then added to the crystallization well containing the crystal. The final concentration of the small molecules was approximately 20 mM. The soaking duration was set to at least 24 h. After soaking, all crystals were immediately flash-frozen in liquid nitrogen and stored after being treated with a cryoprotectant solution containing 25% glycerol and crystallization buffer.

### Data collection and structure determination

Crystal diffraction data were collected at beamlines BL18U1 and BL19U1, and processed using the deployed automated pipelines, primarily utilizing xia2-xds and AUTOPROC for initial processing (23, 24, 25). For cases where data processing failed, HKL3000 was used for supplementary processing (26). After data collection, an automated pipeline was used for full-process structure determination. Preliminary structure solutions were obtained using Dimple in the CCP4 suite with the PDB entry 7OFU serving as the initial model for molecular replacement (27). Subsequently, manual inspection of the electron density maps, particularly those containing small molecules, was conducted. Iterative refinement and model building methods were applied to further optimize the structures. The refinement process was carried out using PHENIX and Coot (28, 29). The statistics for data collection and refinement are summarized in Statistical Parameters Table.

### Molecular dynamics (MD) simulations

SWISS-MODEL was initially employed to reconstruct missing amino acid residues and side chains in the crystal structures, covering residues numbered from 2 to 314. For molecular dynamics (MD) simulations, both monomeric and crystallographically assembled dimeric PLpro–ligand complexes were prepared. The PLpro dimer structures were generated according to the crystallographic symmetry operations of the corresponding crystal structures. Before initiating molecular dynamics (MD) simulations, protein–ligand complexes were prepared using the Amber99SB force field for proteins and the General Amber Force Field (GAFF) for ligands. Protein structures were processed with the GROMACS (https://manual.gromacs.org/) utility pdb2gmx, utilizing the Amber99SB force field and the SPC/E water model (30). Ligand topology files were generated with ACPYPE, applying GAFF parameters. The protein–ligand complexes were solvated in SPC/E water molecules within a cubic simulation box with a minimum distance of 1.0 nm between the solute and the box boundaries. Sodium and chloride ions were added to neutralize the systems and achieve an ionic concentration of 0.15 M. Periodic boundary conditions were applied in all directions. Energy minimization was carried out using the steepest descent method until the maximum force was reduced below 1000 kJ/mol/nm. Following energy minimization, the systems underwent equilibration in two stages: first, a 100 ps NVT equilibration at 300 K, and subsequently a 100 ps NPT equilibration at 300 K and 1 bar. After equilibration, production MD simulations were performed under NPT conditions for 100 ns with a 2 fs integration timestep. Long-range electrostatic interactions were calculated using the Particle Mesh Ewald (PME) method (31), and all hydrogen-containing bonds were constrained using the LINCS algorithm (32). After completion of the MD simulations, trajectories were processed by removing periodic boundary effects. Protein structural stability was evaluated by calculating the RMSD of protein Cα atoms. Ligand RMSD values were calculated after aligning the trajectories based on the Cα atoms of PLpro, allowing evaluation of ligand positional stability within the binding pocket during the simulations. The RMSF values of protein Cα atoms were further calculated to characterize residue-level flexibility and local conformational changes. RMSF analyses were performed using the equilibrated trajectories from the last 50 ns of each 100 ns production simulation.

### PLpro enzymatic activity assay

PLpro activity was monitored using the substrate peptide-AMC (RLLRGG-AMC, Cat. No. HY-P4388A, MCE). Experiments were performed in 96-well black non-binding plates (Cat. No. 3925, Corning) with a final reaction volume of 120 μl. The assay buffer contained 50 mM Tris, pH 7.5, 0.1 mg/ml BSA. 40 μl of diluted compound solutions were added to 96-well plates first, then PLpro was added to the plates at a final concentration of 50 nM. After mixing, incubate the mixture at room temperature for 30 min. Enzyme reactions were initiated with 40 μl of peptide-AMC (final 20 μM) dissolved in the above assay buffer. Upon addition of the peptide substrate, incubating the mixture at room temperature for 5 min. The fluorescence signals were monitored at 360 nm (excitation) and 460 nm (emission) in a biotek Synergy Neo microplate reader.

## Data availability

The atomic coordinates have been deposited in the Protein Data Bank (PDB accession code: 21FX 21GA 9M8Z 9M90 9M91 9M92 9M93 9M95 9M96 9M97 9M99 9M9A 9M9B 9M9C 9M9J 9M9K 9MAF 9MAJ 9MAL 9MAM 9M9L 9MAC 9MAB 9MA9 9MAA. All other data are contained within the article.

## Supporting information

This article contains supporting information (12, 33).

## Conflict of interest

The authors declare that they have no conflicts of interest with the contents of this article.
