# Supplementary material for: Discovery of novel druggable binding pockets on SARS-CoV-2 PLpro via fragment screening and insights for structure-based inhibitor design
Source: J Biol Chem. 2026 Aug 14;302(10):113453. doi: 10.1016/j.jbc.2026.113453 (PMC13578403; doi:10.1016/j.jbc.2026.113453)
Supplement: Supplementary file 1 [file mmc1.docx]

**Supplementary Figure 1. Structural superimposition of the wild-type protein****(PDB ID:7NFV) (1)and the C111S mutant. The wild-type structure and the C111S mutant structure were aligned using 269 Cα atoms. The resulting Cα RMSD was 0.286 Å, indicating minimal overall structural deviation caused by the C111S mutation.**

**Supplementary Figure 2**. Electron density map of the fragment compound at Site 1. Contour levels: 2Fo–Fc at 1.0σ (blue), Fo–Fc at 3.0σ (green).

**Supplementary Figure 3**. Electron density map of the fragment compound at Site 2. Contour levels: 2Fo–Fc at 1.0σ (blue), Fo–Fc at 3.0σ (green).

**Supplementary Figure 4**. Electron density map of the fragment compound at Site 3. Contour levels: 2Fo–Fc at 1.0σ (blue), Fo–Fc at 3.0σ (green).

**Supplementary Figure 5**.Electron density map of the fragment compound at Site 4. Contour levels: 2Fo–Fc at 1.0σ (blue), Fo–Fc at 3.0σ (green).

**Supplementary Figure 6**. Electron density map of the fragment compound at other Site. Contour levels: 2Fo–Fc at 1.0σ (blue), Fo–Fc at 3.0σ (green).

**Supplementary Figure 7**. Summary of the crystallographic fragment-screening datasets for PLpro. Box plots super-impose a scatter plot depict distributions of key diffraction-quality metrics, including Rmerge, resolution (Å), and completeness (%). Outliers are omitted for clarity.

**Supplementary Figure 8**. Electron density map of the fragment compound at Site1 and site2. Contour levels: 2Fo–Fc at 1.0σ (blue), Fo–Fc at 3.0σ (green).

**Supplementary Figure9.** Multiple sequence alignment of PLpro from MERS-CoV, SARS-CoV, and SARS-CoV-2. Conserved amino acids are indicated by darker shading, while divergent residues appear in lighter colors. Key residue differences at positions 164, 273, and 302 are highlighted with red boxes.

**Supplementary Figure 10.** Interactions between PLPro-C111S and Fragment Molecules at other sites. Hydrogen bonds are represented by yellow dashed lines and hydrophobic interactions are represented by gray dashed lines**.**

**Supplementary Figure 11.** Crystal packing arrangement of PLpro highlighting potential dimer interfaces and fragment-binding sites. Interactions between monomers (green and cyan) suggest close proximity of fragments bound to Site 1 and Site 3. This indicates the possibility of linking these fragments into a stable dimeric form. Enlarged panels (right) show detailed views of fragments simultaneously occupying Sites 1 and 3 (upper), as well as the unique binding mode of Frag124 at Site 4 near the dimer interface (lower).

**Supplementary Figure 12.** Structural basis and molecular dynamics assessment of the Frag124-associated PLpro dimer. **(A)** Structural comparison of the PLpro dimer with the SARS-CoV-2 PLpro–ISG15 complex (PDB 7RBS)(2). **(B)** Superposition of snapshots obtained from the molecular dynamics simulation of the PLpro dimer at 0 ns, 20 ns, 40 ns, 60ns,80 ns, and 100 ns. **(C)** Cα RMSD of the PLpro dimer with and without Frag124 in 0 -100 ns. **(D)** Snapshots of the fraf124 with PLPro dimer at 0 ns, 20 ns, 40 ns, 60ns,80 ns, and 100 ns. The initial interfacial binding cavity of Frag124 is indicated by the red dashed box. **(E)** RMSD of the Frag124 in PLpro dimer in 0 -100 ns**.**

**Supplementary Figure 13.** Enzymatic inhibition of the 23 structurally characterized fragments against PLpro. All fragments were tested at a final concentration of 1 mM under identical assay conditions. Data are presented as mean ± S.D. from three independent experiments.

**Supplementary Table 1. Statistical parameters for data collection and refinement.** The statistics for data collection and refinement are provided in **Statistical_Parameters_Table.xlsx.** Values in parentheses correspond to the highest-resolution shell. Rmerge is defined as Σ|I − ⟨I⟩| / ΣI, where I is the observed intensity and ⟨I⟩ is the average intensity of symmetry-related reflections. CC1/2 represents the correlation coefficient between two random half datasets.

1.	Srinivasan, V., Brognaro, H., Prabhu, P. R., de Souza, E. E., Günther, S., Reinke, P. Y. A., Lane, T. J., Ginn, H., Han, H., Ewert, W., Sprenger, J., Koua, F. H. M., Falke, S., Werner, N., Andaleeb, H., Ullah, N., Franca, B. A., Wang, M. Y., Barra, A. L. C., Perbandt, M., Schwinzer, M., Schmidt, C., Brings, L., Lorenzen, K., Schubert, R., Machado, R. R. G., Candido, E. D., Oliveira, D. B. L., Durigon, E. L., Niebling, S., Garcia, A. S., Yefanov, O., Lieske, J., Gelisio, L., Domaracky, M., Middendorf, P., Groessler, M., Trost, F., Galchenkova, M., Mashhour, A. R., Saouane, S., Hakanpää, J., Wolf, M., Alai, M. G., Turk, D., Pearson, A. R., Chapman, H. N., Hinrichs, W., Wrenger, C., Meents, A., and Betzel, C. (2022) Antiviral activity of natural phenolic compounds in complex at an allosteric site of SARS-CoV-2 papain-like protease. *Commun Biol* **5**, 805.

2.	Wydorski, P. M., Osipiuk, J., Lanham, B. T., Tesar, C., Endres, M., Engle, E., Jedrzejczak, R., Mullapudi, V., Michalska, K., Fidelis, K., Fushman, D., Joachimiak, A., and Joachimiak, L. A. (2023) Dual domain recognition determines SARS-CoV-2 PLpro selectivity for human ISG15 and K48-linked di-ubiquitin. *Nature Communications* **14**, 2366.
